# AiKPro: deep learning model for kinome-wide bioactivity profiling using structure-based sequence alignments and molecular 3D conformer ensemble descriptors

**DOI:** 10.1038/s41598-023-37456-8

**Published:** 2023-06-24

**Authors:** Hyejin Park, Sujeong Hong, Myeonghun Lee, Sungil Kang, Rahul Brahma, Kwang-Hwi Cho, Jae-Min Shin

**Affiliations:** 1 AZothBio Inc., Rm. DA724 Hyundai Knowledge Industry Center, Hanam-si, Gyeonggi-do Republic of Korea; 2 School of Systems Biomedical Science, Soongsil University, Seoul, Republic of Korea

**Keywords:** Data mining, High-throughput screening, Machine learning, Protein function predictions, Virtual drug screening, Computational biology and bioinformatics, Drug discovery, Structural biology

## Abstract

The discovery of selective and potent kinase inhibitors is crucial for the treatment of various diseases, but the process is challenging due to the high structural similarity among kinases. Efficient kinome-wide bioactivity profiling is essential for understanding kinase function and identifying selective inhibitors. In this study, we propose AiKPro, a deep learning model that combines structure-validated multiple sequence alignments and molecular 3D conformer ensemble descriptors to predict kinase-ligand binding affinities. Our deep learning model uses an attention-based mechanism to capture complex patterns in the interactions between the kinase and the ligand. To assess the performance of AiKPro, we evaluated the impact of descriptors, the predictability for untrained kinases and compounds, and kinase activity profiling based on odd ratios. Our model, AiKPro, shows good Pearson’s correlation coefficients of 0.88 and 0.87 for the test set and for the untrained sets of compounds, respectively, which also shows the robustness of the model. AiKPro shows good kinase-activity profiles across the kinome, potentially facilitating the discovery of novel interactions and selective inhibitors. Our approach holds potential implications for the discovery of novel, selective kinase inhibitors and guiding rational drug design.

## Introduction

Protein kinases are pivotal regulators of various cellular processes, including cell growth, differentiation, migration, metabolism, and apoptosis^1^. By phosphorylating substrate proteins, kinases modulate signaling pathways that play crucial roles in the initiation and progression of human diseases such as cancer, inflammation, cardiovascular diseases, and neurodegenerative disorders^2,3^. Due to their central roles in cellular regulation, protein kinases have emerged as attractive therapeutic targets, sparking extensive research efforts to discover and develop selective and potent kinase inhibitors. Since the approval of the first kinase inhibitor, imatinib, in 2001, over 70 kinase inhibitors have been granted regulatory approval for clinical use^4^. In recent years, kinase inhibitors have consistently accounted for approximately 20% of all newly approved drugs^5–8^, with a significant proportion being multi-target inhibitors^9^. Multi-target drugs aim to block multiple signaling pathways or protein–protein interactions, potentially providing greater therapeutic efficacy and overcoming the limitations of single-target inhibitors^10^.

However, the development of selective kinase inhibitors remains challenging due to the high degree of structural and sequence similarity of the kinase domain, which is conserved across the protein kinase family^11^. This structural conservation often results in limited selectivity and off-target effects, which can compromise the safety and efficacy of kinase inhibitors. One notable example is sorafenib, a multi-kinase inhibitor targeting RAF kinases and VEGFR. While initial clinical trials showed significant improvement in patients with thyroid cancer, further analysis revealed that its failure to improve overall survival was primarily due to off-target effects, including the inhibition of other kinases involved in regulating cell growth and survival. This resulted in toxicity, such as severe hand-foot skin reactions, which limited its use and efficacy^12^. Therefore, accurately predicting the binding profile of kinase inhibitors is crucial to avoid cross-reactivity and ensure successful drug development.

Various computational methods have been employed for kinase inhibitor profiling, including ligand-based approaches relying on the chemical properties of small molecules and structure-based approaches incorporating protein structure information^13–20^. Machine learning-based predictive models have shown considerable success in profiling the bioactivity of kinase inhibitors. Several machine learning algorithms, such as support vector machines (SVM)^21^, naïve Bayes (NB)^22^, random forests^23^, and neural networks^24,25^, have been applied to this task, using different types of input features, such as molecular fingerprints^26^, protein sequences^27^, ligand simplified molecular-input line-entry system (SMILES) strings^27^, drug-target similarity matrices^28^, or molecular graphs^29^. Recent advances in deep learning have further expanded the repertoire of computational methods available for kinase inhibitor profiling. Deep learning architectures, such as graph neural networks (GNNs)^30^, Recurrent Neural Networks (RNN)^31^, and attention mechanisms^32^, have been employed to predict kinase inhibitor selectivity and potency with increasing accuracy. These methods have shown promise in dealing with complex and high-dimensional input features, such as protein sequences, molecular structures, and protein–ligand interaction patterns.

Despite the success of these machine learning and deep learning approaches, there remain several limitations associated with the use of traditional input features and algorithms for kinase inhibitor profiling. One major limitation is that most of these methods do not adequately account for the three-dimensional (3D) structure of proteins and compounds, which is a critical factor influencing their interactions and binding affinities. To address this limitation, more advanced descriptors, such as molecular 3D conformer ensemble descriptors (3CED), have been proposed to better represent the structurally dynamic state of molecules and improve the accuracy of interaction prediction between small molecules and proteins^33^. In addition to 3D conformer ensemble representation of compounds, incorporating protein structure information into kinase inhibitor profiling models is crucial for achieving high predictive performance^31,34–36^. One approach in this regard could be the use of structure-validated multiple sequence alignments (svMSA), which provide a detailed view of the kinase active site and account for the conservation and variation in protein structures across the kinome^37^. By combining svMSA with 3CED, we believe it is possible to develop more accurate and robust deep learning models for kinase profiling that can better capture the intricate relationships between kinase and compound structures and their interaction patterns.

In this study, we introduce a new deep learning architecture named AiKPro that integrates 3D-structural information from both kinases and compounds. We use svMSA and 3CED to obtain the kinase and compound information, respectively, for kinome-wide bioactivity profiling. AiKPro leverages the attention mechanism to effectively capture the complicated interaction patterns between kinases and compounds, resulting in better predictions of kinase-ligand binding affinities and identification of selective kinase inhibitors. We performed several experiments to evaluate the performance of AiKPro. These included: (1) conducting fivefold cross-validation on the training set, (2) validating the performance of the consensus model on independent test sets, (3) comparing the results with those of molecular docking methodologies, (4) assessing the model’s performance on untrained kinases and compounds, and (5) evaluating the model’s applicability for kinome-wide profiling.

## Methods

### Data processing

**Figure 1:**
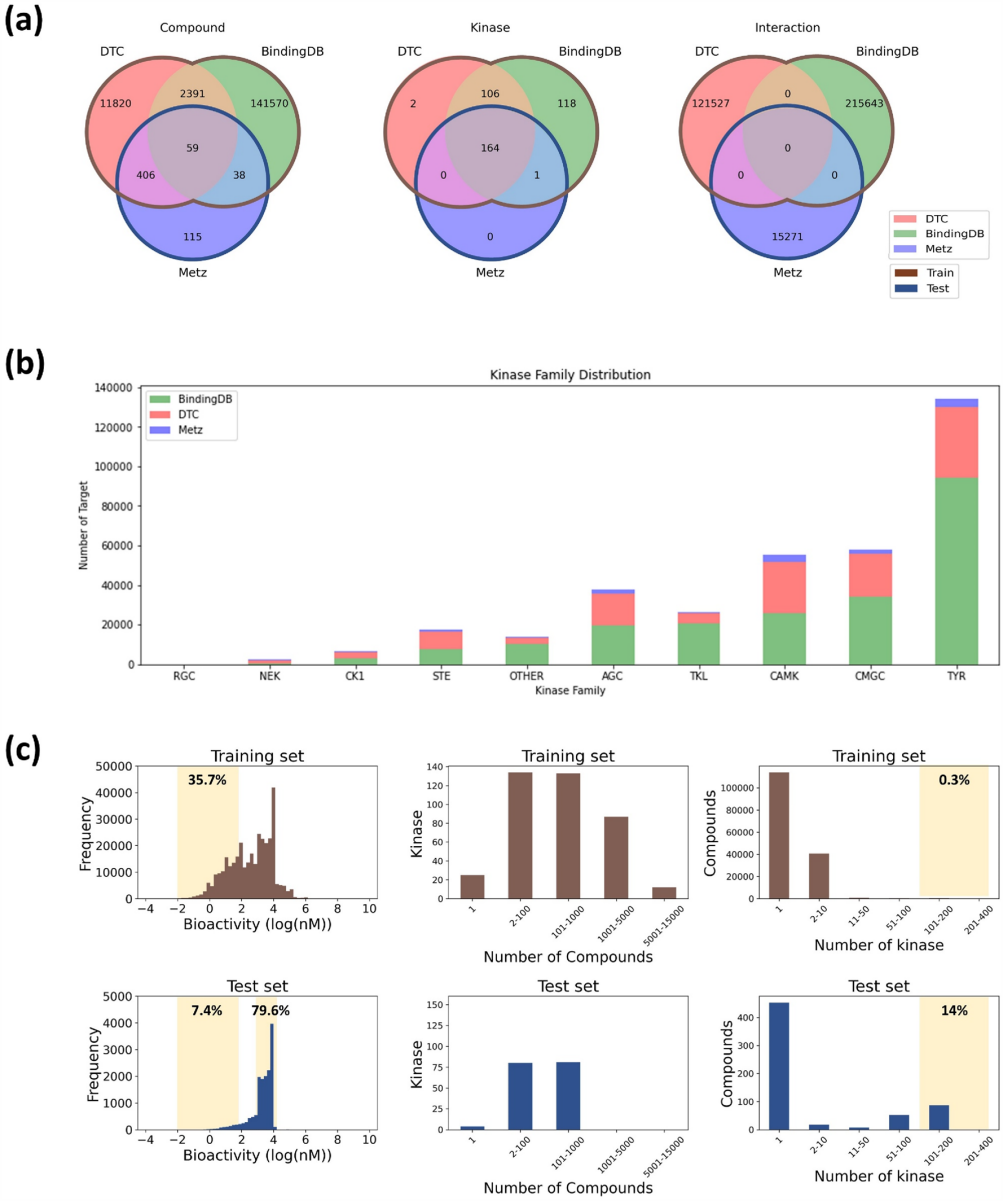
Overview of the AiKPro dataset. The figure presents (**a**) the connections among compounds, kinases, and bioactivity within the three reference databases, (**b**) the distribution of data points per kinase group in each reference database, and (**c**) the count of data for bioactivity, kinases, and compounds in the training and test sets.

To develop a deep learning approach capable of kinome-wide profiling, we collected a large amount of bioactivity data have been collected from several publicly databases including BindingDB^38^, Drug Target Commons (DTC)^39^, and the Metz dataset^40^. To ensure high data quality, we filtered out all data points lacking bioactivity values or target kinase information. Additionally, we curated the dataset by matching all kinase targets to svMSA kinase and removing any non-experimental values with IC_50_ or Ki. To eliminate bias, we used the mean activity value for each kinase-compound pair when multiple values were available. Bioactivity was defined as log (IC_50_ or K_i_). For model validation, we designated BindingDB and DTC as a training set, and Metz dataset as the test set. Further details about the dataset in the study are shown in Fig. 1 and Table 1. Table 1 provides a summary of all the datasets used, Fig. S1 provides a comprehensive overview of each preprocessing step, and Table S1 lists all the 391 kinase targets included in the training set of AiKPro.

**Table 1:** Summary of AiKPro dataset and strict split subsets.

Dataset name	Train/test	Data source	Kinases	Compounds	Bioactivity
AiKPro dataset	Train	BindingDB, DTC	391	156,284	337,171
	Test	Metz	165	618	15,271
Strict split for docking	Train	BindingDB, DTC	391	156,284	337,171
	Test	BindingDB, DTC, Metz	6	148	563
Strict split for kinases	Train	BindingDB, DTC	388	156,202	335,648
	Test	Metz	3	1,505	1,522
Strict split for compounds	Train	BindingDB, DTC	391	155,781	287,493
	Test	Metz	165	618	15,271

To compare our deep learning model with structure-based docking studies, we created a docking dataset by searching the Protein Data Bank (PDB) for kinase-compound interactions. We obtained 3D structure data for six kinases (ABL1, CDK9, MELK, AKT1, LCK, and CLK2) complexed with ligands, and selected 563 compounds for the test set. The Fig. S2 provides more information on the quantity of each type of data.

To evaluate the robustness and performance of the AiKPro model, we created separate evaluation datasets, as summarized in Table 1. The first dataset, called ‘Strictly split for Kinases’, aimed to assess the ability of the model to predict bioactivity values for kinases that were not included in the training process. Three kinases (HCK, CLK1 and PRKCE) from different kinase groups (CMGC, AGC and TYR) were selected based on their large number of bioactivity data points (400–600) and wide range of bioactivity values (variance > 1). This dataset consisted of 1522 bioactivity values for the three kinases, which served as the test set, while the remaining 335,648 bioactivity data points were used for model training. The second dataset, called “Strict split for Compounds”, aimed to evaluate the performance of the model in predicting bioactivity values for compounds not present in the training set. This dataset contained all bioactivity data for 618 compounds from the Metz dataset, which served as an external test set. The bioactivity values of these compounds were excluded from the training set of the AiKPro dataset. The resulting training set contained 287,494 bioactivity data points, covering 391 kinases and 155,781 compounds. Using these evaluation datasets, we performed a comprehensive assessment of the performance of the AiKPro model and its applicability in diverse scenarios.

### Input representations

The kinase representations in this study were encoded using one-hot encoding techniques in three different ways: Identifier, svMSA^AS^, and svMSA. Identifier is a one-hot encoding of the kinase entry, while svMSA^AS^ represents the active site sequences using a sequence variation of the multiple sequence alignment method^41^. The svMSA representation is derived from the full sequence variation of the multiple sequence alignment method, specifically from the kinase domain sequence alignment. These methods resulted in embeddings of size (1, 391) for Identifier, (29, 21) for svMSA^AS^, and (2218, 21) for svMSA, where the 21-vector includes 20 standard amino acids and the “− “ insertion code.

For compound representation, RDKit software^42^ was used to extract molecular features from isomeric and canonicalized SMILES representations. The molecular descriptors were determined using three primary components: (a) 208 physicochemical properties (PCPP), including topological and topochemical descriptors, partition coefficient (LogP), molar refractivity (MR) using Crippen’s approach^43^, and Lipinski parameters^44^; (b) 167 MACCS (Molecular ACCess System) fingerprints (MCFP)^45^, and (c) Morgan fingerprints (MGFP)^46^ with radii of 2, 3, and 4 bonds, respectively, each comprising 256, 512, and 1024 bits. These MGFPs were concatenated, resulting in a feature vector of 1792 bits in length.

Incorporating 3D descriptors into our model is crucial for capturing the influence of different features of interacting sites on kinase selectivity. To achieve this, we used a 3D pharmacophore signature factory, which generates up to 16 conformers per compound to represent their structural diversity. The 3D pharmacophore signature factory was generated using minimal features defined in RDKit and accounts for all feature types in 3D space, including stereoisomerism. We calculated 3348 pharmacophore signatures for each conformer, divided into three bins (0–3 Å, 3–6 Å, and 6–9 Å). The final 3CED vector was obtained by calculating the mean values of the feature vectors from all conformers of the compound, representing their different spatial arrangements.

### Model architecture

We developed six different models to investigate whether incorporating both kinase and compound structural information could improve model performance. These models used a basic set of compound descriptors, including PCPP, MCFP, and MGFP. These models are as follows:

Model 1–1: Identifier + PCPP + MCFP + MGFP,

Model 1–2: Identifier + PCPP + MCFP + MGFP + 3CED,

Model 2–1: svMSA^AS^ + PCPP + MCFP + MGFP,

Model 2–2: svMSA^AS^ + PCPP + MCFP + MGFP + 3CED,

Model 3–1: svMSA + PCPP + MCFP + MGFP,

Model 3–2 (AiKPro): svMSA + PCPP + MCFP + MGFP + 3CED.

Models 1-, 2-, and 3-* use one-hot encoding for kinase entries, active site (svMSA^AS^), and full svMSA of kinase, respectively. Model *-2, unlike model *-1, includes 3CED. AiKPro (Model 3–2) incorporates kinase and compound structural information using svMSA and 3CED.

**Figure 2:**
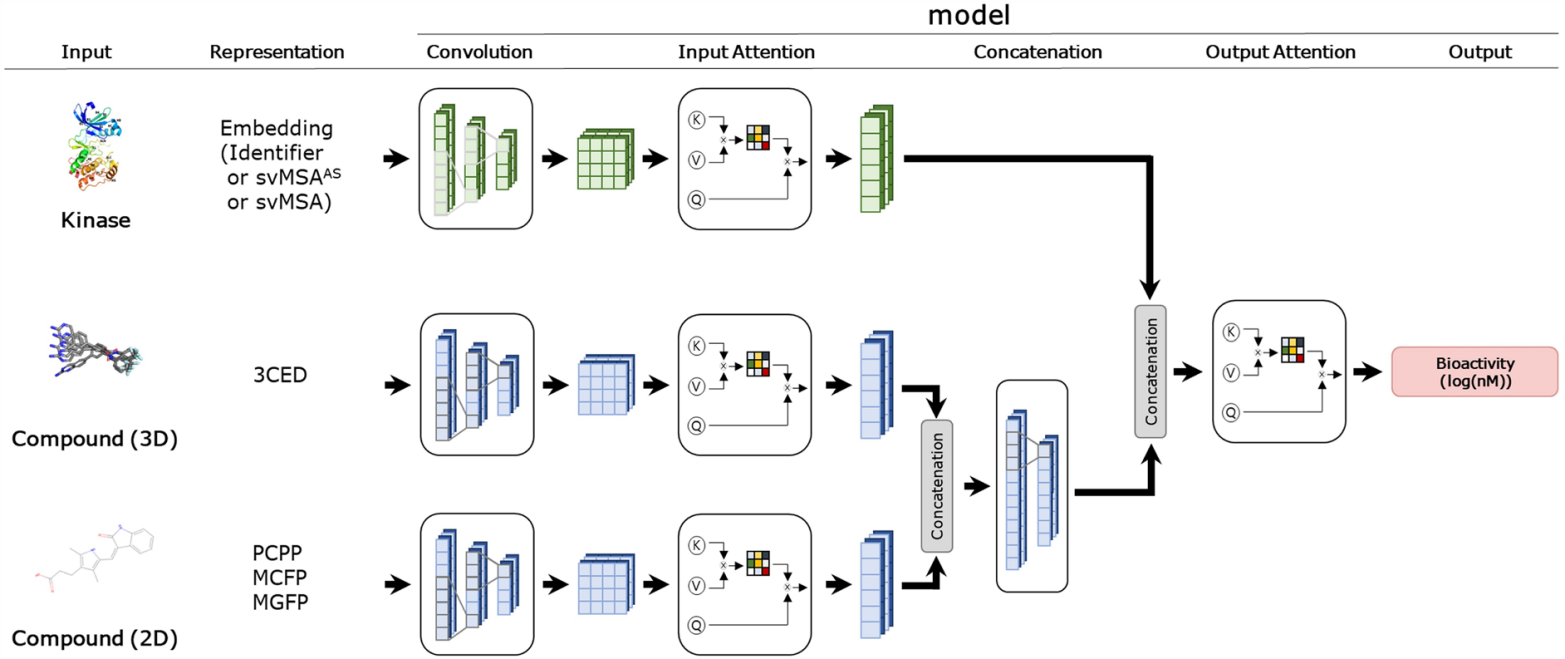
Schematic representation of model architectures. The diagram shows the kinase and compound representations used in the models. Kinase input includes kinase entry, svMSA^AS^, and svMSA. Compound (3D) represents the 3CED descriptor from the 3D conformers of compound, while Compound (2D) consists of three descriptors (PCPP, MCFP, and MGFP) derived from the compound’s 2D structure.

Each of the models in this study employs five different blocks: Convolution, Input Attention, Concatenation, Output Attention, and Output blocks. The Convolution and Input Attention blocks are responsible for extracting feature tensors from the input representation. These feature tensors are then merged in the Concatenation block and used for bioactivity prediction through Output Attention block and the Output block, respectively (see Fig. 2).

### Model training and performance evaluation

To evaluate and compare the performance of the six models, we employed a similar approach. We used the mean square error (MSE) as the loss function for training and conducted training for a maximum of 3000 epochs with an early stopping criterion of 100 epochs based on the validation loss. During the training process, we used a batch size of 512 was used during the training process, and more details on the training configuration can be found in Table S2. We trained one model for each architecture and evaluated each model through fivefold cross validation, comparing the mean and error range of the following metrics: Pearson correlation coefficient (PCC), R-squared (R^2^), root mean square error (RMSE) and mean absolute error (MAE). Finally, we performed prediction and validation on the test set using a consensus method that averages the predictions of the five models, as shown in Fig. S3.

To perform the docking study, we selected PDB structures based on the best resolution available in the RCSB PDB (www.rcsb.org), including 4WA9, 6FYL, 3MY1, 2OFV, 4BKY, and 3OCB. Molecular self-docking studies of these compounds were performed using AutoDock Vina, one of the most widely cited open-source molecular docking programs^47^. A standard rigid-receptor setup was used, utilizing the Vina and AutoDock 4.2 scoring functions. The parameters used in the docking study are listed in Table S3. All target and ligand structures were prepared as flexible, following the protocol outlined in the literature reference for AutoDock Vina^48,49^. The results of the two scoring functions using AutoDock Vina docking were compared with the predictions by AiKPro. To compare the predicted bioactivity of AiKPro with the AutoDock Vina score, ROC analysis was performed. Various metrics including Precision, Recall, F1-Score, Area Under the Curve (AUC), and Accuracy were evaluated to assess the performance of both methods. The experimental bioactivity data was categorized into active or inactive based on predetermined cut-off values of 0.1 µM, 1 µM, and 5 µM. In order to facilitate a fair comparison, min–max scaling was applied to normalize the predicted bioactivity values of AiKPro and the AutoDock Vina score.

For evaluation of kinase profiling studies, we used odds ratio (OR)^50^. We used the same OR-value calculation strategy to other workers’ kinase profiling studies^13^ where 1 µM is the cutoff value for ‘positive’ or ‘negative’ bioactivity and OR is calculated as following equation:

$$OR=\frac{\frac{{N}_{GP}}{{N}_{OP}}}{\frac{{N}_{GN}}{{N}_{ON}}}$$

Here ${N}_{GP}$ represents the number of positive interactions of an inhibitor within a specific group, and ${N}_{OP}$ represents the number of positive interactions with a kinase outside a specific group. Similarly, ${N}_{GP}$ and ${N}_{OP}$ are defined as the number of negative interactions for each. If the OR value is significantly higher than 1.0, it can be inferred that the kinases inhibited by a particular inhibitor are enriched within the group in question, indicating a degree of selectivity for that inhibitor^13^. We used OR values and KinMap^51^, a web-based visualization tool, to evaluate and visualize the results of kinase profiling studies. By using OR-values and KinMap, we can better see the selectivity of kinase inhibitors and their potential applications.

## Results

### Bioactivity dataset distribution

The curated kinase dataset consisted of a training set of 156,284 compounds and a test set of 618 compounds. While a significant portion of compounds in the test set (81%) overlapped with those in the training set, it is noteworthy that the validation was specifically designed to assess the distinct interaction between compounds and targets, with no overlap between training and test sets, as shown in Fig. 1a. In particular, there are 165 common kinases in the training and test sets, and 115 unique compounds are selected only in the test set. Figure 1a shows a Venn diagram highlighting the distribution of compounds in the training and test sets, including the number of unique compounds present in each set.

In Fig. 1b, it can be seen that there are similar distributions of kinase groups between the two sets, with a few exceptions, such as the RGC, NEK, and TYR groups. Interestingly, the test set had a lower percentage of TYR (27.1%) compared to the training set (38.6%). On the other hand, the AGC group showed a higher percentage in the test set (16.2%) than in the training set (10.5%). Furthermore, the NEK, CK1, STE, AGC, and CAMK kinase groups had a higher distribution in the test set than in the training set. Overall, these results suggest that the test set was sufficiently diverse and independent from the training set.

Figure 1c shows the distribution of bioactivity values in the training and test sets. Notably, the training set has a higher proportion (35.7%) of interaction data with bioactivity values between − 2 and 2 (top left). Conversely, the test set shows predominantly bioactivity values between 3 and 4, with only a small percentage (7.4%) falling in the – 2–2 range (bottom left). Regarding the distribution of kinases, the majority of kinases in the AiKPro dataset are associated with bioactivities between 2 and 1000 compounds (middle). Among the kinases in the training set, 99 have bioactivity values for more than 1000 compounds (top center), while the kinases in the test set show interactions with a maximum of 562 compounds (bottom center). Notably, approximately 73% of the compounds in both sets have data corresponding to only one kinase. In addition, only 0.3% of the data in the training set have interactions with more than 100 kinases, whereas more than 14% of the data in the test set have interactions with more than 100 kinases. This analysis of the ‘AiKPro Dataset’ underlines the different characteristics observed between the training and test sets, highlighting the suitability of the dataset for accurately predicting bioactivity across a wide range of chemical spaces.

### Performance of AiKPro

We constructed 6 different models using different input representations of kinase and compound. These models were based on a combination of convolutional neural networks (CNN) and attention mechanisms. To avoid data bias, fivefold cross-validation was performed on the training set. Among these models, AiKPro was found to perform the best, with a PCC (Pearson correlation coefficient) value of 0.90 (± 0.01) and an R^2^ value of 0.79 (± 0.01) during the fivefold cross-validation process. However, it is important to note that the performance differences between the models were not significant, as shown in Fig. S4.

**Figure 3:**
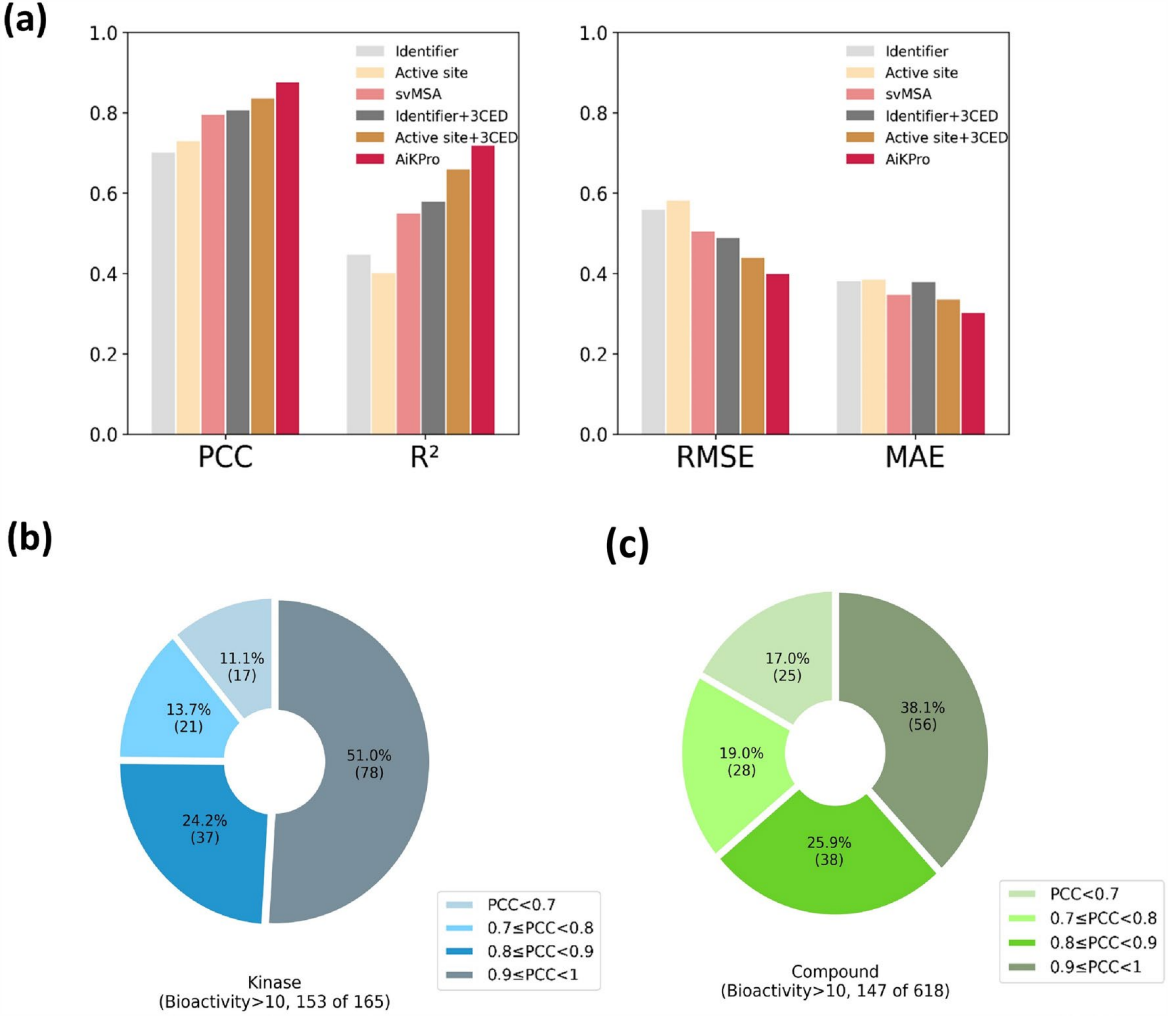
Test set performance evaluation. The figure displays (**a**) the performance metrics for each model on the test set, and the PCC distribution for (**b**) kinases and (**c**) compounds using the AiKPro.

The test set results of the five consensus models showed significant performance differences between the six different architectures. In particular, for the input representation of compounds using PCPP, MGFP, and MCFP, Models 1–2, 2–2, and 3–2 using 3CED performed better than Models 1–1, 2–1, and 3–1 which did not use 3CED. Figure 3 and Table S4 show that the use of 3CED improved PCC values and metrics such as R^2^, RMSE, and MAE. This highlights the importance of 3CED in improving model performance. Furthermore, the PCC values increased sequentially for Models 1–2, 2–2, and 3–2 using Identifier, svMSA^AS^, and svMSA representations with 3CED. Among the models, AiKPro using svMSA and 3CED showed the best performance, indicating that the use of svMSA and 3CED leads to more accurate predictions of kinase bioactivity compared to models using traditional kinase and molecular representations.

To assess the robustness of model, kinases and compounds with more than 10 interaction data points in the test set were selected and PCC values were calculated. The results, as shown in Fig. 3b,c, show that AiKPro performed well, with PCC values greater than 0.7 for more than 80% of the selected kinases and compounds. This indicates that the model is quite robust and can accurately predict bioactivity values for a wide range of kinases and compounds.

To evaluate the performance of AiKPro in predicting kinase inhibitory activity and kinase-drug interactions, we performed benchmark tests. Given the heterogeneity of kinase activity datasets, direct model comparisons are challenging. To mitigate this, we evaluated the performance of AiKPro using the well-established Metz dataset^40^, which has been used in previous studies^52^. The results, shown in Table S5, consistently demonstrate the superior performance of AiKPro across multiple evaluation metrics, confirming its efficacy in accurately predicting kinase-ligand interactions. These results provide robust evidence for the reliability and efficacy of AiKPro as an advanced tool in this field.

### Evaluation studies on untrained data

Predicting the bioactivity of untrained kinases or compounds is a critical yet challenging task in drug discovery and development. In this study, we evaluated the performance of our deep learning models in predicting the bioactivity of untrained kinases using the “Strict split for kinases” dataset shown in Table 1. The dataset contains three subsets, each consisting of one kinase from a different group, to evaluate the model's ability of the model to accurately predict bioactivity values for untrained kinases. We completely removed the corresponding kinase data from the training set, and the test set contained only that specific kinase data. The consensus prediction results for the independently trained models showed excellent performance, with PCC values greater than 0.70 for each of the three kinases evaluated: 0.74 for CLK1 (P49759, CMGC), 0.71 for PRKCE (Q02156, AGC), and 0.82 for HCK (P08631), as summarized in Table 2. These results demonstrate the potential of our models to accurately predict bioactivity values for untrained kinases, highlighting their potential in drug discovery and development.

**Table 2:** Evaluation metrics for AiKPro model on strict split subsets.

Test data	Count	Variance	PCC	R^2^	RMSE	MAE
Strict split for kinases	1522	1.50	0.71	0.47	0.89	0.72
CLK1	450	1.02	0.69	0.29	0.85	0.73
PRKCE	488	1.71	0.71	0.48	0.94	0.78
HCK	584	1.59	0.77	0.55	0.85	0.65
Strict split for compounds	15,271	0.56	0.87	0.73	0.39	0.29
Strict split for docking	563	0.63	0.89	0.78	0.38	0.28

In addition, we evaluated the model’s performance of the model in predicting the bioactivity of untrained compounds using the “Strict split for compounds” dataset, as shown in Table 1. Our primary objective was to determine whether the model could accurately profile novel, previously untrained molecules. From the 618 compounds in the test set, we selected 147 compounds with 10 or more kinases and evaluated the prediction results for each, as shown in Fig. 3. The consensus model incorporating both svMSA and 3CED showed strong performance with a PCC value of 0.87 and an R^2^ value of 0.73 for all untrained compounds, indicating the model’s ability to accurately predict the bioactivity of untrained compounds. These results suggest that our deep learning models can be an effective tool in drug discovery and development by more accurately predicting bioactivity scores for both untrained kinases and compounds.

### Kinase class selectivity of AiKPro

**Figure 4:**
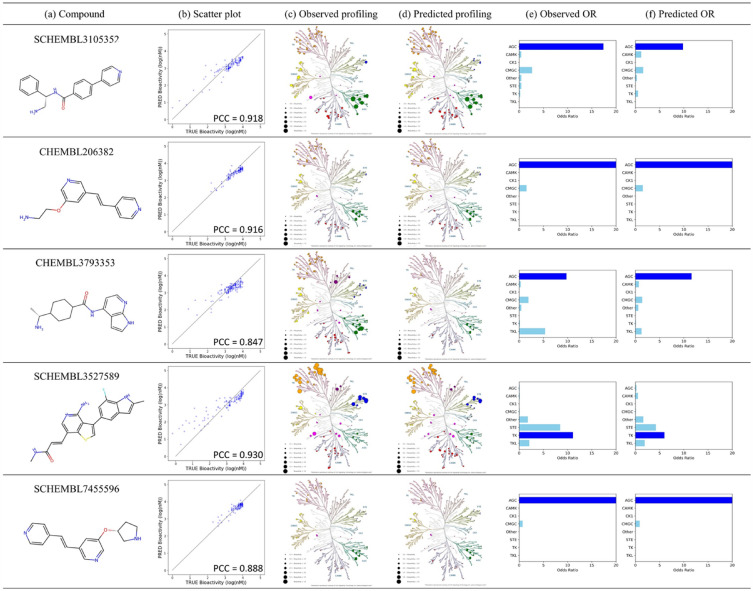
Comparison of AiKPro-predicted and experimental bioactivity values. Column (**a**) represents the test compound. Column (**b**) presents a scatter plot illustrating the correlation between the bioactivity values calculated by AiKPro and the corresponding experimental values. Columns (**c**) and (**d**) show the kinome trees calculated using the experimental and predicted bioactivities of the test compounds, respectively. Similarly, columns (**e**) and (**f**) exhibit the OR plots for the experimental and predicted values, respectively.

The results of the odds ratio (OR) value assessment for the kinase classes demonstrate that AiKPro, trained with both svMSA and 3CED, is an effective tool for kinase profiling. For the calculation of odds ratio (OR) values, a cut-off value of 3.0 (1uM) was used to define ‘positive’ and ‘negative’ interaction. Out of the initial 618 compounds in the test set, we selected 103 that showed activity in both classes for calculating OR values. As Fig. 1c demonstrates that a significant proportion of these 618 compounds had limited binding activities with 10 or fewer kinases, restricting their usefulness for comparative kinase class analysis. Additionally, the requirement for values to be in both ‘positive’ and ‘negative’ classes led us to choose a subset of 103 compounds meeting this criterion. The experimental and predicted bioactivity values of these compounds are summarized in Supplementary Information Sheets 1 and 2. Out of 103 compounds, 79 were accurately assigned to their corresponding kinase group with a high ratio of valid bioactivity, consistent with previous studies using OR to indicate kinase group selectivity, such as the study by Li et al.^15^, In addition, Fig. 4 shows five compounds with high group specificity, further highlighting the ability of the model to accurately predict bioactivity values and its potential for drug discovery and development. In Fig. 4, Column (a) represents the test compounds, while Column (b) displays a scatter plot and Pearson’s correlation coefficient, illustrating the correlation between the predicted and experimental values. Columns (c) and (d) present the kinome trees calculated using the experimental and predicted bioactivities of the test compounds, respectively. Similarly, columns (e) and (f) exhibit the OR plots for the experimental and predicted values, respectively. For more detailed understanding of the kinome trees, please refer to Fig. S5 in the Supplementary Information. These findings demonstrate that AiKPro not only predicts the bioactivity profiles of each of the 391 kinases but also the kinase class profiles.

### Comparison to molecular docking study

To compare the performance of AiKPro with a commonly used molecular docking tool, we used AutoDock Vina to dock 563 bioactivity data points and compared the results with those generated by AiKPro. Our analysis showed that AiKPro showed a superior performance in predicting the binding affinity of compounds for selected kinases, as evidenced by its higher correlation for experimental binding affinity on a logarithmic scale (PCC = 0.89) compared to AutoDock Vina (PCC = 0.36). Figure S2 shows that AiKPro consistently showed higher correlation values for all kinases tested, including CLK1, PRKCE, and HCK. For example, AiKPro achieved PCC values of 0.92, 0.91, and 0.91 for CLK1, PRKCE, and HCK, respectively, whereas AutoDock Vina achieved PCC values of only 0.47, 0.41, and 0.34 for the same kinases, respectively.

Furthermore, we performed ROC analysis to evaluate the predictive performance of AiKPro compared to the AutoDock Vina score for bioactivity prediction. The analysis included the use of different cut-off values for experimental bioactivity. The results, presented in Table S6 and Fig. S6, consistently show that AiKPro outperforms AutoDock Vina with significantly higher area under the curve (AUC) values at the three cutoffs of 0.1 µM, 1 µM and 5 µM. AiKPro achieves an AUC of 0.98 at the 0.1 µM cutoff, while AutoDock Vina achieves an AUC of 0.76. Similarly, at the 1 µM and 5 µM cutoffs, AiKPro shows superior AUC values of 0.90 and 0.89, respectively, compared to AutoDock Vina's AUC values of 0.64 and 0.67. These results highlight the significant advantage of AiKPro over AutoDock Vina in terms of predictive accuracy, particularly at the stringent 0.1 µM cut-off. These results underscore the effectiveness of AiKPro in accurately predicting bioactivity for kinase-ligand interactions.

While it's important to note that our analysis was limited to a specific set of test cases and cannot be generalized to all scenarios, the results do suggest that AiKPro has the potential to be a more reliable tool for predicting binding affinity values, especially for kinases. In addition, the high correlation values obtained by AiKPro suggest the advantages of using a deep learning approach over traditional molecular docking techniques, and further research may be warranted to explore these advantages in more detail.

## Discussion

In this study, we present AiKPro, a novel deep learning model for kinase profiling that integrates svMSA and 3CED. Our unique approach to kinase and compound representation has demonstrated improved prediction performance compared to conventional methods (see Fig. 3). AiKPro’s attention-based deep learning architecture was shown to be superior to existing methods, as evidenced by the high Pearson correlation coefficients obtained for both the test set and untrained compounds.

Our results show that AiKPro effectively captures complicated kinase-compound interaction patterns, resulting in a robust and extensible model. The high Pearson correlation coefficients obtained in both the unknown kinase evaluation study (PCC > 0.7) and the unknown compound evaluation study (PCC > 0.87) support the notion that svMSA and 3CED capture crucial kinase structural features and potential ligand structural information that are important for kinase selectivity.

Integration of data on kinase-ligand interaction from different source is a challenging task, due to the wide range of experiment types and definitions of activities, which could lead to significant systematic errors in results^54,55^. To address this challenge, previous studies have employed classification models, rather than regression models, or trained each data source separately^16,18,56,57^. On the contrary, our study used both IC50 and Ki values as targets for bioactivity, which are commonly used in the literature as measures of inhibitor potency and can provide a more comprehensive coverage of the dataset. Although mixing IC50 and Ki values can introduce potential errors, we attempted to address this by curating and filtering the datasets used in our study and averaging values from multiple assays where available. In addition, we used deep learning methods that can learn from a diverse set of data and identify patterns from multiple sources, which we believe is a strength of our study. We anticipate that the large quantity and diversity of data used in our model will increase its robustness, enabling for more precise predictions of kinase-ligand interactions.

Furthermore, AiKPro demonstrated superior scoring performance when compared to AutoDock Vina, a widely used traditional docking tool. AutoDock Vina mainly scores poses using pairwise interatomic distances and includes a repulsive parabolic function, making it sensitive to bumps and strains^48^. In contrast, AiKPro uses 3D structure information from svMSA and 3CED, allowing approximate binding affinity predictions with significantly reduced computational demands. This allows for rapid profiling of large numbers of molecules, making AiKPro a more efficient tool for virtual screening and drug discovery. Despite the significant advantages, it is important to recognize its limitations and the challenges associated with in silico kinase profiling. An important issue is the applicability of the model to kinases with mutations, which is particularly relevant in kinase-related drug discovery^58^. In addition, it is unclear whether the model can be effectively extended to a wider range of compound structures. Addressing these challenges provides opportunities for future research to refine the model and enhance its capabilities, ultimately increasing the potential applicability of AiKPro in drug discovery.

To further extend the scope of AiKPro, future research should focus on incorporating additional data sources, investigating less studied kinases, and improving the model's ability to account for kinase point mutations. The continued development of AiKPro, combined with the integration of new data and insights, will enable researchers to more effectively navigate the complex landscape of kinase drug discovery more effectively, leading to the development of innovative treatments for various diseases.

Recently, a deep learning-based method combined with molecular dynamics (MD) has successfully identified three inhibitors for a new cancer drug target, TIPE2^59^. Additionally, the integration of deep learning and molecular docking simulations has led to the identification of potentially effective FDA-approved drugs for repurposing against SARS-CoV-2^60^. Deep learning techniques have also been employed to predict quantum mechanical (QM) descriptors, resulting in accurate estimations of molecular properties and reactivity^61^. The developed model has shown reasonable predictions for various ground-state QM properties. Similarly integrating AiKPro with such in-silico techniques may offer a comprehensive understanding of kinase-ligand interaction, elucidation their crucial role in drug discovery and specificity^62,63^. This synergistic approach can provide a more accurate and holistic view of kinase inhibitor interactions, guiding the design of targeted and effective therapeutics.

As in silico kinase profiling methods continue to evolve, researchers can explore new techniques and methods to further improve the performance of models such as AiKPro. These may include the improved representation of protein–ligand interactions and the development of more accurate scoring functions^64,65^. In addition, the availability of kinase and compound datasets is likely to continue to increase, allowing for continuous refinement and incorporation of new knowledge to ensure that the model remains at the cutting edge of kinase drug discovery research^66^. Another area of potential improvement is the incorporation of dynamic information on protein and ligand conformations. While our model considers the conformational ensemble of small molecules, it does not take into account the dynamic nature of protein structures, which can be critical in determining the selectivity and potency of kinase inhibitors. Future research can investigate the integration of dynamic information on protein and ligand conformations to further improve the predictive accuracy of AiKPro.

Furthermore, the application of AiKPro can be extended beyond the scope of kinase drug discovery to explore the selectivity and specificity of other protein families and signaling pathways involved in various diseases. By expanding the applicability of the model, researchers can gain a more complete understanding of the underlying molecular mechanisms and develop targeted therapies for a wider range of diseases, ultimately benefiting patients and healthcare providers. In summary, the development of AiKPro represents a significant advance in the field of in silico kinase profiling and provides a valuable tool for drug discovery efforts. Ongoing improvements and refinements to the model, combined with the integration of new data and techniques, will further enhance its utility in guiding the design of novel, targeted therapies^53–55^.

In summary, AiKPro represents a significant advance in the field of in silico kinase profiling offering the ability to efficiently identify selective kinase inhibitors, prioritize candidates for experimental validation, and inform rational drug design strategies, ultimately streamlining the drug development process^56^. Moreover, the accurate prediction of kinase inhibitor selectivity profiles can guide the optimization of lead compounds, minimizing adverse side effects and enhancing their therapeutic potential^67^. With the potential to significantly impact the field of kinase drug discovery, AiKPro has opened up new avenues for large-scale high-throughput kinome profiling and the development of more effective and selective kinase-targeted therapies for various diseases.

As the field continues to evolve, further advancements in computational methods and the integration of new knowledge will undoubtedly improve the predictive accuracy and applicability of models such as AiKPro, paving the way for more effective and targeted therapies for a wide range of diseases. The development of AiKPro is just the beginning of what is achievable in the field of in silico kinase profiling, and future research will focus on addressing the model's limitations, refining its capabilities, and expanding its scope. The continued improvement and application of AiKPro and similar models will advance the field of kinase drug discovery forward, ultimately leading to the discovery of novel therapeutics with improved efficacy and safety profiles.

## Supplementary Information

Supplementary Information 1.

Supplementary Information 2.

## Data Availability

The source of the BindingDB dataset can be found at https://www.bindingdb.org/. The source of the DTC can be found at https://drugtargetcommons.fimm.fi/. The source of the Metz Dataset can be found at https://doi.org/10.1038/nchembio.530.

## Abbreviations

PDB: Protein Data Bank
3CED: Molecular 3D conformer ensemble descriptor
SMILES: Simplified molecular-input line-entry system
svMSA: Structure-validated multiple sequence alignment
svMSA^AS^: Active site positions of svMSA
PCPP: Physicochemical properties
logP: Partition coefficient
MR: Molar refractivity
MACCS: Molecular ACCess system
MCFP: MACCS fingerprint
MGFP: Morgan fingerprint
2D: Two-dimensional
3D: Three-dimensional
GNN: Graph neural network
RNN: Recurrent neural network
CNN: Convolutional neural network
PCC: Pearson correlation coefficient
R^2^: R-squared
MAE: Mean absolute error
MSE: Mean square error
RMSE: Root mean square error
OR: Odd ratio

## Code availability

The source code of the AiKPro methods will be made available after publication on a GitHub repository at: https://github.com/AzothBio/AiKPro/.
