# Supplementary material for: AiKPro: deep learning model for kinome-wide bioactivity profiling using structure-based sequence alignments and molecular 3D conformer ensemble descriptors
Source: Sci Rep. 2023 Jun 24;13:10268. doi: 10.1038/s41598-023-37456-8 (PMC10290719; doi:10.1038/s41598-023-37456-8)
Supplement: Supplementary file 1 — Supplementary Information 1. [file 41598_2023_37456_MOESM1_ESM.docx]

**Supplementary Information**

**AiKPro: Deep Learning Model for Kinome-Wide Bioactivity Profiling Using Structure-based Sequence Alignments and Molecular 3D Conformer Ensemble Descriptors**

Hyejin Park^1^, Sujeong Hong^1^, Myeonghun Lee^1^, Sungil Kang^1^, Rahul Brahma^2^, Kwang-Hwi Cho^2^, Jae-Min Shin^1^*

^1^AzothBio, Rm. DA724 Hyundai Knowledge Industry Center, Hanam-si, Gyeonggi-do, Republic of Korea

^2^School of Systems Biomedical Science, Soongsil University, Seoul, Republic of Korea

**Supplementary Figure S1. Schematic flow of the data curation process, which involves data acquisition, data cleaning, and data splitting into training and test sets.**

**
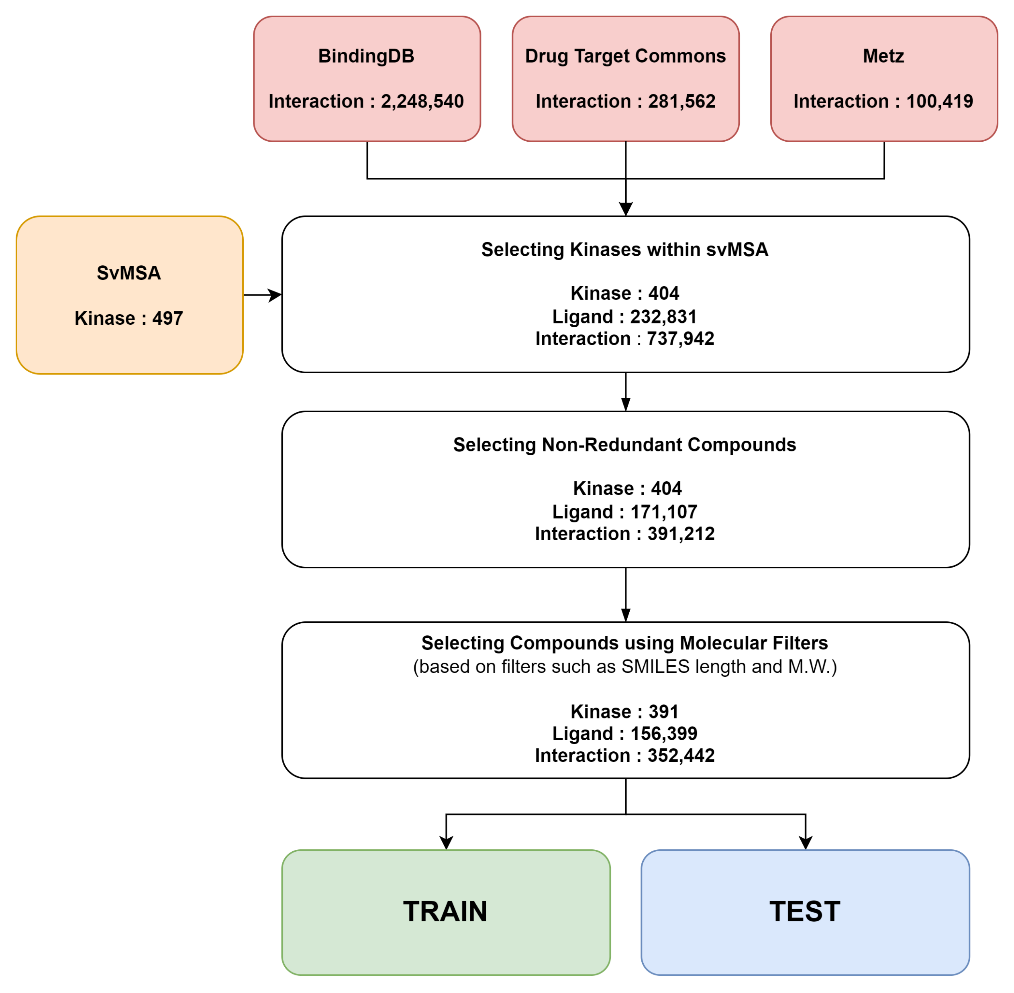
**

**Supplementary Figure S2. Comparison of AiKPro predictions and molecular docking scores.**

**
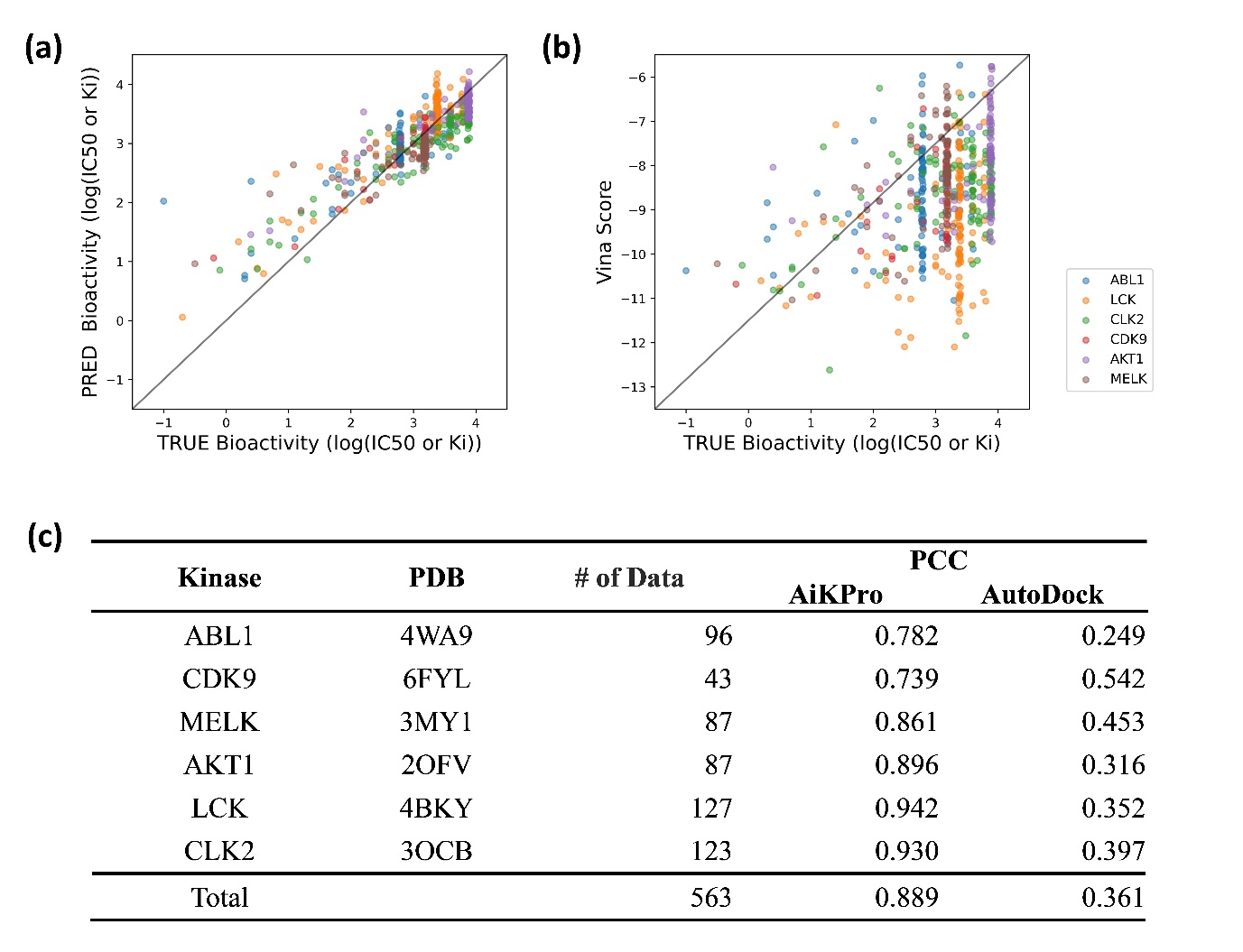
**

Scatter plots, (a) and (b) illustrates the correlation between the observed log10(IC50/Ki) (M) and the predicted values of AiKPro and molecular docking score, respectively. Table (c), summarizes the Pearson correlation coefficient (PCC) values for six kinases, comparing AiKPro and molecular docking methods.

**Supplementary Figure S3.** **Visualization of the 5-fold cross-validation process and ensemble prediction method employed by AiKPro.**


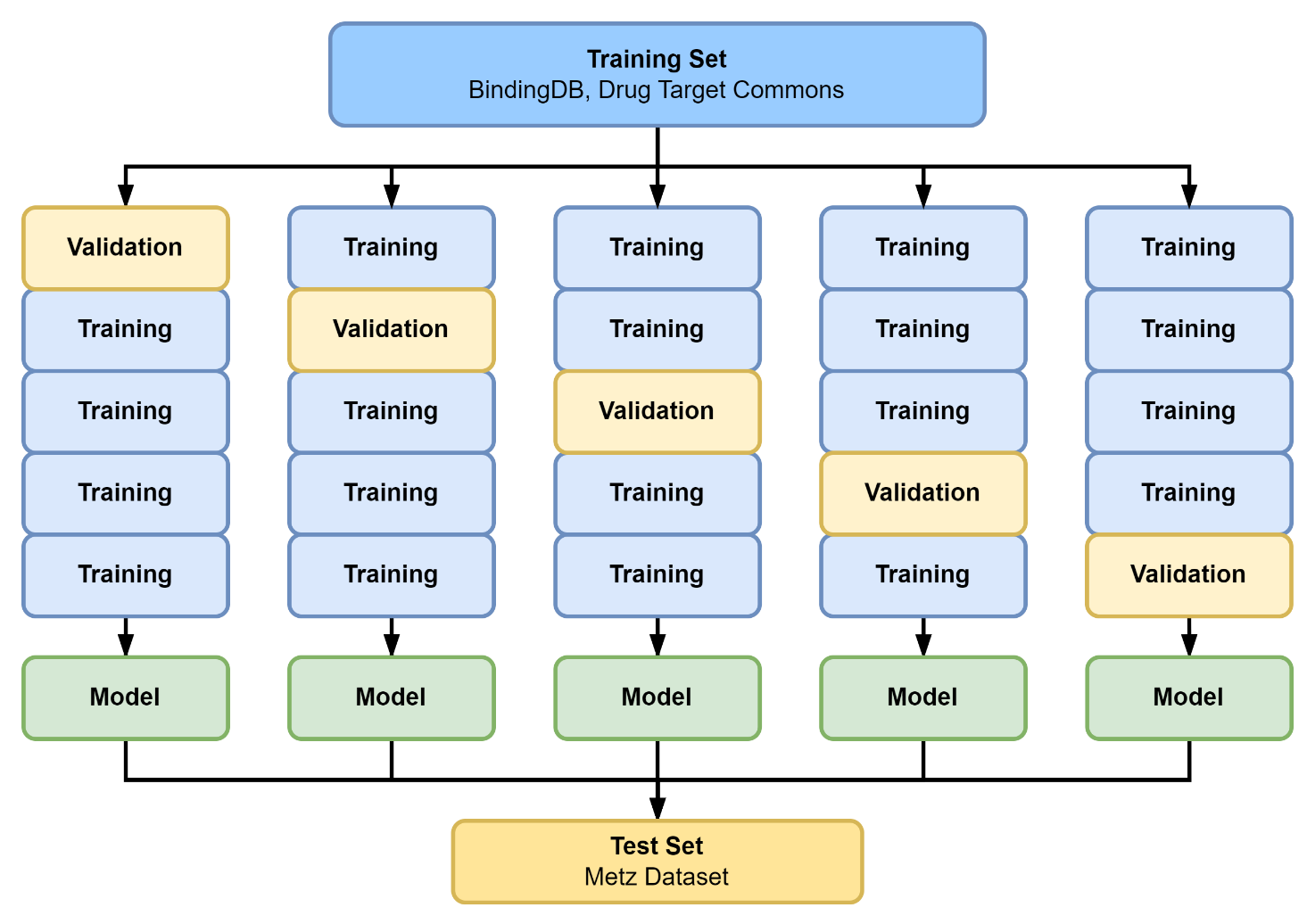


**Supplementary Figure S4. Learning curve and scatter plot of actual-predicted bioactivity for each architecture.**

**
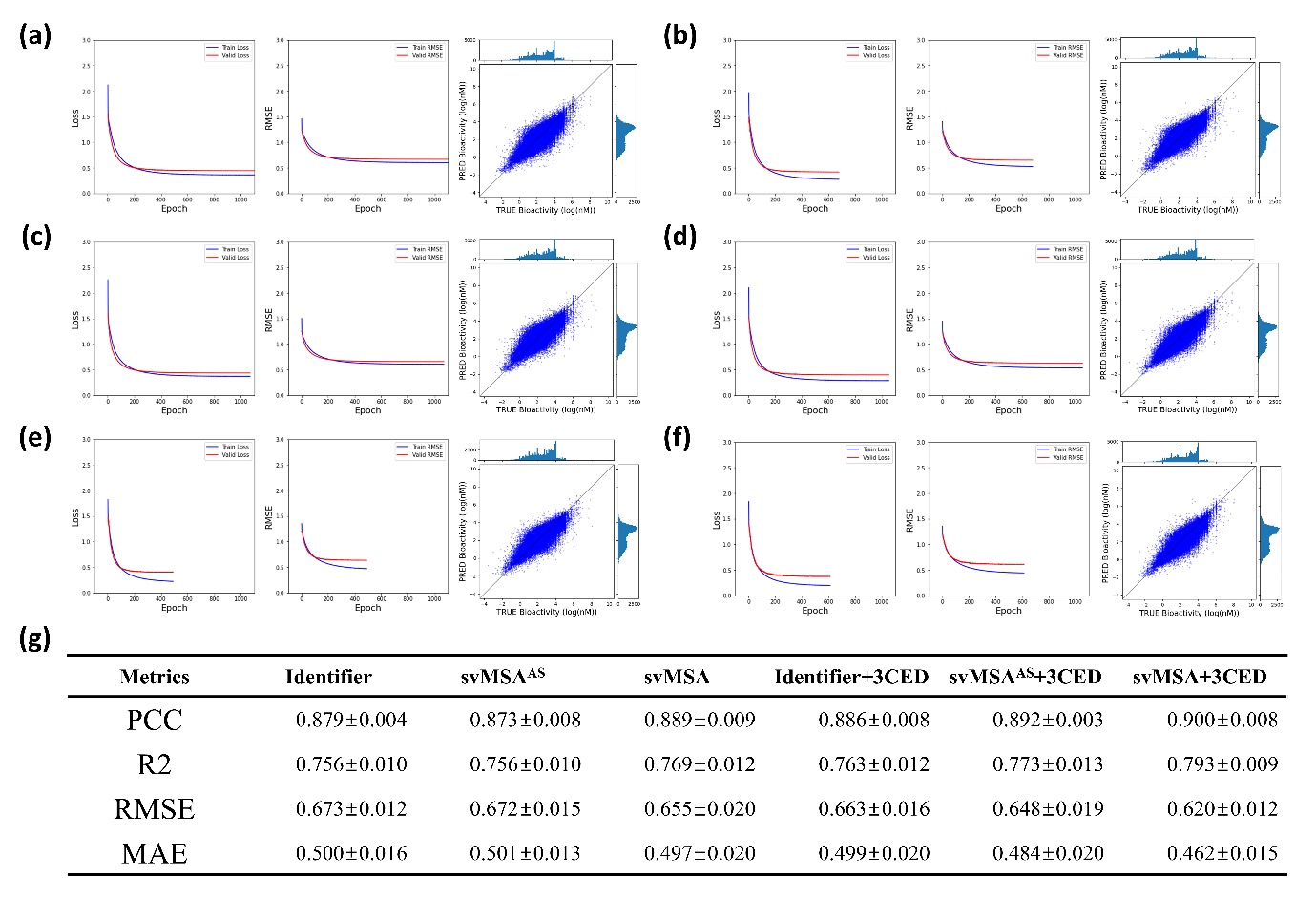
**

The models using Identifier, svMSA^AS^, and svMSA are illustrated in panels (a), (c), and (e), respectively. Panels (b), (d), and (f) show models where 3CED was used for (a), (c), and (e), respectively. The 5-fold cross-validation performance is summarized in panel (g).

**Supplementary Figure S5. Human Kinome Trees for the five representative compounds.**

The figure provides a detailed visualization of the bioactivity of the compounds, as shown in Figure 4(c) and (d). The size of each circle corresponds to the strength of the bioactivity, with larger circles indicating higher levels of bioactivity. The bioactivity is categorized into seven levels based on the size of the circles, providing a comprehensive view of the bioactivity distribution across the kinome. Importantly, these figures show the agreement between experimental observations and AiKPro predictions of the bioactivity distribution.

**Supplementary Figure S6. Evaluation of AiKPro and AutoDock Vina Performance using Receiver Operating Characteristic (ROC) Curves at Various Active Cutoffs.**

**
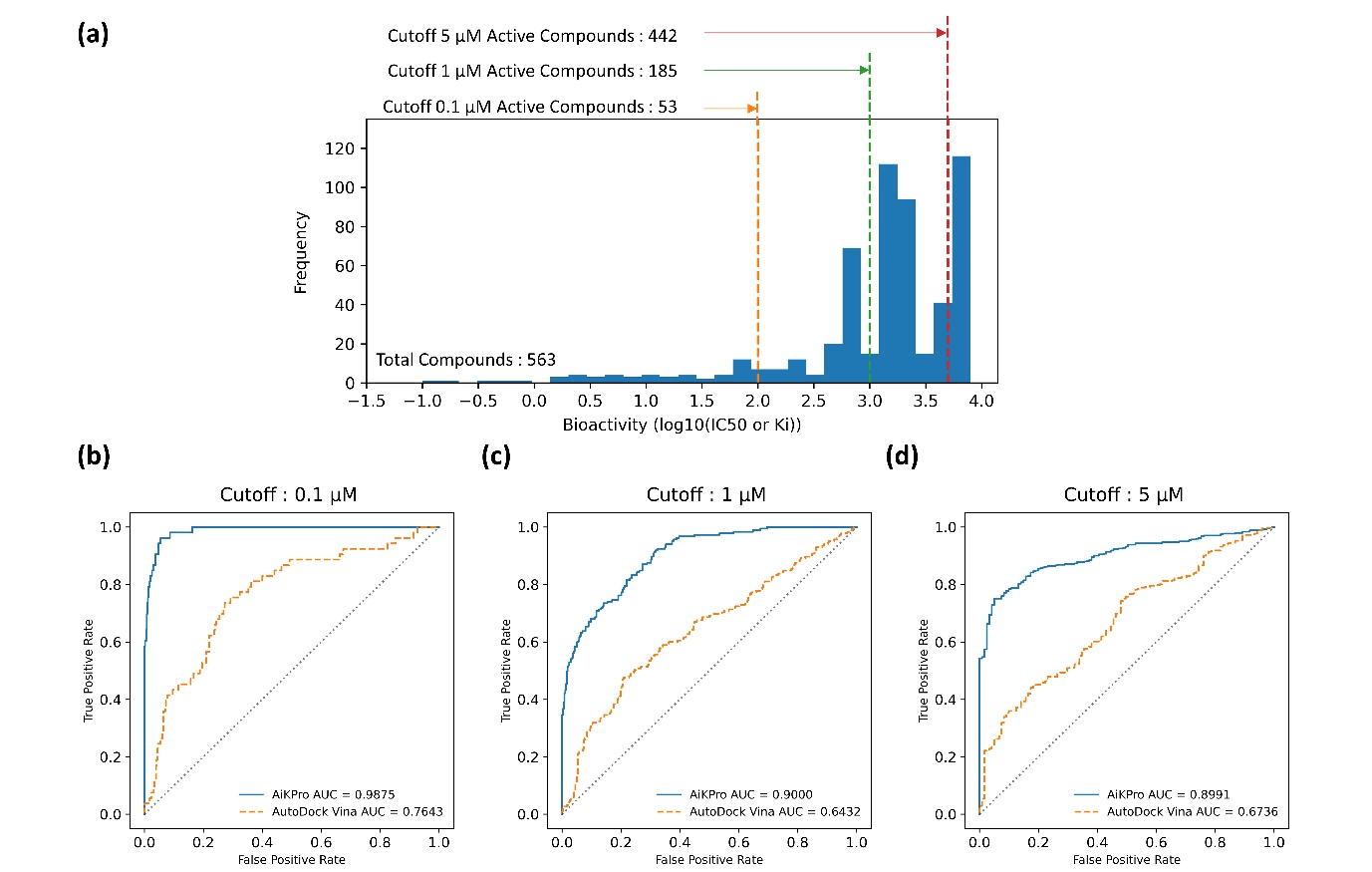
**

Panel (a) displays the distribution of values in the test set, with the majority falling between 2.5 and 4. The OR value cutoffs of 0.1 μM, 1 μM, and 5 μM are represented by the yellow, green, and red dashed lines, respectively. The corresponding ROC curves for each cutoff are shown in panels (b), (c), and (d), respectively.

**Table S1. List of 391 Kinase Names and UniProt Entries for AiKPro.**

| **Name** | **Entry** | **Name** | **Entry** | **Name** | **Entry** | **Name** | **Entry** |
| --- | --- | --- | --- | --- | --- | --- | --- |
| KDR | P35968 | PEAK1 | Q9H792 | SRPK3 | Q9UPE1 | MET | P08581 |
| MAPK8 | P45983 | MKNK1 | Q9BUB5 | CAMK2A | Q9UQM7 | ERBB2 | P04626 |
| SYK | P43405 | TSSK1B | Q9BXA7 | WNK2 | Q9Y3S1 | MAP4K4 | O95819 |
| MAPK9 | P45984 | TSSK6 | Q9BXA6 | MAP3K2 | Q9Y2U5 | MAST3 | O60307 |
| CSNK1E | P49674 | CDK19 | Q9BWU1 | STK38L | Q9Y2H1 | NUAK1 | O60285 |
| CDK8 | P49336 | STK31 | Q9BXU1 | SIK3 | Q9Y2K2 | IGF1R | P08069 |
| MAPKAPK2 | P49137 | MKNK2 | Q9HBH9 | TNIK | Q9UKE5 | PRKD3 | O94806 |
| CLK1 | P49759 | MAP3K14 | Q99558 | DAPK2 | Q9UIK4 | CDK14 | O94921 |
| CLK2 | P49760 | PIK3R4 | Q99570 | TAOK2 | Q9UL54 | MAP3K6 | O95382 |
| CLK3 | P49761 | PKMYT1 | Q99640 | TLK1 | Q9UKI8 | OXSR1 | O95747 |
| CSNK1D | P48730 | MAP3K5 | Q99683 | IRAK4 | Q9NWZ3 | RIPK2 | O43353 |
| CSNK1A1 | P48729 | EIF2AK1 | Q9BQI3 | NEK6 | Q9HC98 | BUB1 | O43683 |
| MAP2K4 | P45985 | VRK1 | Q99986 | CSNK1G1 | Q9HCP0 | MAP3K7 | O43318 |
| MAP2K3 | P46734 | MAP3K3 | Q99759 | BMP2K | Q9NSY1 | DYRK3 | O43781 |
| TGFBR1 | P36897 | NTRK2 | Q16620 | STK36 | Q9NRP7 | RET | P07949 |
| ACVRL1 | P37023 | MAP3K11 | Q16584 | LATS2 | Q9NRM7 | NTRK1 | P04629 |
| TGFBR2 | P37173 | DDR2 | Q16832 | SNRK | Q9NRH2 | STK16 | O75716 |
| CSK | P41240 | PHKG1 | Q16816 | PAK6 | Q9NQU5 | STK17B | O94768 |
| FLT3 | P36888 | MAPKAPK3 | Q16644 | DYRK4 | Q9NR20 | PAK3 | O75914 |
| ACVR1B | P36896 | MAPK14 | Q16539 | CDK12 | Q9NYV4 | STK10 | O94804 |
| BMPR1A | P36894 | CAMK4 | Q16566 | PLK2 | Q9NYY3 | EGFR | P00533 |
| MATK | P42679 | AAK1 | Q2M2I8 | MAP3K20 | Q9NYL2 | KIT | P10721 |
| PRKCI | P41743 | TNNI3K | Q59H18 | STK32B | Q9NY57 | DAPK3 | O43293 |
| TEC | P42680 | CDKL4 | Q5MAI5 | BTK | Q06187 | PRKCB | P05771 |
| TXK | P42681 | LRRK2 | Q5S007 | PRKCD | Q05655 | PDGFRB | P09619 |
| MAP3K8 | P41279 | SBK1 | Q52WX2 | PRKCZ | Q05513 | RAF1 | P04049 |
| ZAP70 | P43403 | MYLK3 | Q32MK0 | PTK2 | Q05397 | ARAF | P10398 |
| ABL2 | P42684 | MAP3K19 | Q56UN5 | CDK5 | Q00535 | FGR | P09769 |
| GRK6 | P43250 | PIM3 | Q86V86 | CDK6 | Q00534 | WEE2 | P0C1S8 |
| FRK | P42685 | DYRK2 | Q92630 | EPHB4 | P54760 | PDPK1 | O15530 |
| NEK2 | P51955 | HASPIN | Q8TF76 | PRKAA2 | P54646 | PIM1 | P11309 |
| BMX | P51813 | TRIB2 | Q92519 | EPHB3 | P54753 | HCK | P08631 |
| PRKX | P51817 | MYO3B | Q8WXR4 | EPHA5 | P54756 | ROS1 | P08922 |
| NEK3 | P51956 | STK32A | Q8WU08 | EPHB1 | P54762 | LCK | P06239 |
| NEK4 | P51957 | GRK7 | Q8WTQ7 | MAP3K9 | P80192 | ROCK2 | O75116 |
| MAPK12 | P53778 | CDKL2 | Q92772 | CSNK1G2 | P78368 | FYN | P06241 |
| LIMK2 | P53671 | CAMK1D | Q8IU85 | CDKL1 | Q00532 | CDK1 | P06493 |
| PLK1 | P53350 | MAP4K3 | Q8IVH8 | CDK3 | Q00526 | INSR | P06213 |
| LIMK1 | P53667 | HIPK1 | Q86Z02 | CSNK2A1 | P68400 | PRKCG | P05129 |
| DAPK1 | P53355 | MYLK4 | Q86YV6 | SRPK2 | P78362 | YES1 | P07947 |
| MAPK10 | P53779 | VRK2 | Q86Y07 | EPHA4 | P54764 | LYN | P07948 |
| MAP2K6 | P52564 | KSR1 | Q8IVT5 | SIK1 | P57059 | CSF1R | P07333 |
| GSK3B | P49841 | CDKL3 | Q8IVW4 | RIPK4 | P57078 | ERN1 | O75460 |
| GSK3A | P49840 | MAPKAPK5 | Q8IW41 | ACVR1 | Q04771 | ULK1 | O75385 |
| CDK9 | P50750 | BRSK1 | Q8TDC3 | PRKCQ | Q04759 | FES | P07332 |
| IRAK1 | P51617 | STK35 | Q8TDR2 | MST1R | Q04912 | CHEK1 | O14757 |
| BLK | P51451 | NEK9 | Q8TD19 | MAP2K1 | Q02750 | PDGFRA | P16234 |
| CDK7 | P50613 | NEK7 | Q8TDX7 | PRKCE | Q02156 | FER | P16591 |
| MAPK1 | P28482 | MAPK15 | Q8TD08 | CDK16 | Q00536 | PHKG2 | P15735 |
| MARK3 | P27448 | CSNK2A3 | Q8NEV1 | TEK | Q02763 | BRAF | P15056 |
| MAPK3 | P27361 | MYO3A | Q8NEV4 | MAP3K10 | Q02779 | PAK4 | O96013 |
| ACVR2A | P27037 | NEK11 | Q8NG66 | DYRK1A | Q13627 | PRKCA | P17252 |
| GRK2 | P25098 | HIPK4 | Q8NE63 | CAMK2D | Q13557 | ABL1 | P00519 |
| EPHA8 | P29322 | BRSK2 | Q8IWQ3 | IKBKE | Q14164 | PRKACA | P17612 |
| EPHA3 | P29320 | NIM1K | Q8IY84 | PTK6 | Q13882 | PLK4 | O00444 |
| EPHB2 | P29323 | DCLK2 | Q8N568 | BMPR2 | Q13873 | MAP2K7 | O14733 |
| EPHA2 | P29317 | MINK1 | Q8N4C8 | ACVR2B | Q13705 | CIT | O14578 |
| LTK | P29376 | CSNK1A1L | Q8N752 | CDK13 | Q14004 | STK25 | O00506 |
| CDK2 | P24941 | CAMKK1 | Q8N5S9 | PRKG1 | Q13976 | SRC | P12931 |
| PRKCH | P24723 | ULK2 | Q8IYT8 | CAMK1 | Q14012 | AURKA | O14965 |
| MAP2K2 | P36507 | TAOK1 | Q7L7X3 | ROCK1 | Q13464 | IKBKB | O14920 |
| AKT1 | P31749 | NEK8 | Q86SG6 | RIPK1 | Q13546 | INSRR | P14616 |
| AXL | P30530 | MARK2 | Q7KZI7 | CAMK2G | Q13555 | FGFR3 | P22607 |
| WEE1 | P30291 | TLK2 | Q86UE8 | CAMK2B | Q13554 | SGK1 | O00141 |
| TTK | P33981 | STK32C | Q86UX6 | TNK1 | Q13470 | FGFR4 | P22455 |
| GRK4 | P32298 | CDC42BPA | Q5VT25 | PRPF4B | Q13523 | RPS6KB1 | P23443 |
| AKT2 | P31751 | TTBK1 | Q5TCY1 | PKN2 | Q16513 | PRKACG | P22612 |
| FLT4 | P35916 | MAP3K21 | Q5TCX8 | NTRK3 | Q16288 | PRKACB | P22694 |
| GRK5 | P34947 | KSR2 | Q6VAB6 | PKN1 | Q16512 | CHEK2 | O96017 |
| TIE1 | P35590 | DSTYK | Q6XUX3 | MAPK11 | Q15759 | CDC7 | O00311 |
| GRK3 | P35626 | TSSK4 | Q6SA08 | MYLK | Q15746 | FLT1 | P17948 |
| AURKB | Q96GD4 | ULK3 | Q6PHR2 | TESK1 | Q15569 | BMPR1B | O00238 |
| PBK | Q96KB5 | PKN3 | Q6P5Z2 | GRK1 | Q15835 | EIF2AK2 | P19525 |
| CAMKK2 | Q96RR4 | ERN2 | Q76MJ5 | STK11 | Q15831 | FGFR2 | P21802 |
| PASK | Q96RG2 | NEK5 | Q6P3R8 | MELK | Q14680 | ERBB3 | P21860 |
| SRPK1 | Q96SB4 | PNCK | Q6P2M8 | PTK2B | Q14289 | CSNK2A2 | P19784 |
| TESK2 | Q96S53 | TTBK2 | Q6IQ55 | ERBB4 | Q15303 | MAK | P20794 |
| LMTK3 | Q96Q04 | CDC42BPG | Q6DT37 | EPHA7 | Q15375 | CDK11B | P21127 |
| MARK4 | Q96L34 | PIM2 | Q9P1W9 | PRKD1 | Q15139 | EPHA1 | P21709 |
| NEK1 | Q96PY6 | STK26 | Q9P289 | STK38 | Q15208 | FGFR1 | P11362 |
| TSSK3 | Q96PN8 | PAK5 | Q9P286 | DDR1 | Q08345 | CHUK | O15111 |
| TSSK2 | Q96PF2 | TBK1 | Q9UHD2 | TNK2 | Q07912 | DCLK1 | O15075 |
| CAMK1G | Q96NX5 | EPHA6 | Q9UF33 | ITK | Q08881 | MAPK13 | O15264 |
| MAP4K1 | Q92918 | STK39 | Q9UEW8 | TYRO3 | Q06418 | EPHB6 | O15197 |
| SGK3 | Q96BR1 | STK17A | Q9UEE5 | CDK18 | Q07002 | MUSK | O15146 |
| PLK3 | Q9H4B4 | RPS6KB2 | Q9UBS0 | STK4 | Q13043 | GAK | O14976 |
| WNK1 | Q9H4A3 | NLK | Q9UBE8 | MERTK | Q12866 | LATS1 | O95835 |
| HIPK3 | Q9H422 | MARK1 | Q9P0L2 | PAK1 | Q13153 | SGK2 | Q9HBY8 |
| SRMS | Q9H3Y6 | EIF2AK3 | Q9NZJ5 | MAP2K5 | Q13163 | NPR1 | P16066 |
| PRKD2 | Q9BZL6 | DYRK1B | Q9Y463 | PRKAA1 | Q13131 | CDK4 | P11802 |
| STK33 | Q9BYT3 | RIPK3 | Q9Y572 | STK3 | Q13188 | ALK | Q9UM73 |
| WNK3 | Q9BYP7 | MAP4K5 | Q9Y4K4 | MAPK7 | Q13164 |  |  |
| DCLK3 | Q9C098 | CDC42BPB | Q9Y5S2 | PAK2 | Q13177 |  |  |
| NUAK2 | Q9H093 | IRAK3 | Q9Y616 | ILK | Q13418 |  |  |
| HIPK2 | Q9H2X6 | STK24 | Q9Y6E0 | MAP3K1 | Q13233 |  |  |
| TAOK3 | Q9H2K8 | CSNK1G3 | Q9Y6M4 | PRKG2 | Q13237 |  |  |
| SLK | Q9H2G2 | AKT3 | Q9Y243 | DMPK | Q09013 |  |  |
| SIK2 | Q9H0K1 | AURKC | Q9UQB9 | MAP4K2 | Q12851 |  |  |
| MYLK2 | Q9H1R3 | MOK | Q9UQ07 | MAP3K12 | Q12852 |  |  |
| CLK4 | Q9HAZ1 | ICK | Q9UPZ9 | MAP3K4 | Q9Y6R4 |  |  |

**Table S2. Hyperparameters for Training of AiKPro.**

| **Hyperparameter** | **Value** |
| --- | --- |
| Epoch | 3000 |
| Batch size | 512 |
| Learning rate | 0.00001 |
| Patience | 100 |
| Optimizer | Adam |
| Loss Function | MSE |

**Table S3. Parameters for Molecular Docking Study using AutoDock Vina.**

| **Kinase** | | **PDB** | | **Exhaustiveness** | **Grid Box Size (Å)** | | **Grid Box Center** | | | | | |
| --- | --- | --- | --- | --- | --- | --- | --- | --- | --- | --- | --- | --- |
|  |  |  |  |  |  |  | **X** | | **Y** | | **Z** | |
| ABL1 | 4WA9 | | 512 | | 23.389 x 12.803 x 13.22 | -6.829 | | -16.494 | | 12.997 | |  |
| CDK9 | 6FYL | | 512 | | 15.385 x 10.401 x 16.534 | -6.768 | | -17.876 | | 13.858 | |  |
| MELK | 3MY1 | | 512 | | 12.194 x 9.939 x 15.901 | -7.394 | | -15.465 | | 14.754 | |  |
| AKT1 | 2OFV | | 512 | | 22.342 x 17.205 x 12.939 | -9.692 | | -14.739 | | 12.56 | |  |
| LCK | 4BKY | | 512 | | 14.093 x 14.384 x 20.217 | 13.453 | | 10.619 | | 3.713 | |  |
| CLK2 | 3OCB | | 512 | | 15.265 x 14.637 x 16.671 | -7.576 | | -17.055 | | 16.374 | |  |

**Table S4. Performance metrics of AiKPro and other models using different architectures on the test (Metz) dataset.**

| **Metrics** | **PCC** | **R^2^** | **RMSE** | **MAE** |
| --- | --- | --- | --- | --- |
| Identifier | 0.701 | 0.447 | 0.559 | 0.380 |
| Identifier+3CED | 0.806 | 0.578 | 0.488 | 0.378 |
| svMSA^AS^ | 0.729 | 0.400 | 0.582 | 0.384 |
| svMSA^AS^+3CED | 0.836 | 0.658 | 0.439 | 0.334 |
| svMSA | 0.796 | 0.550 | 0.504 | 0.346 |
| svMSA+3CED (AiKPro) | **0.876** | **0.718** | **0.399** | **0.301** |

**Table S5. Performance metrics comparing AiKPro and other models on the Metz dataset.**

|  | | **MSE ± std** | | **CI ± std** | |
| --- | --- | --- | --- | --- | --- |
| DeepDTA | 0.781± 0.060 | | 0.627± 0.011 | |  |
| DeepConvDTI | 0.703± 0.027 | | 0.671± 0.016 | |  |
| TransformerCPI | 1.081± 0.125 | | 0.557± 0.016 | |  |
| GraphDTA | 1.232± 0.094 | | 0.615± 0.010 | |  |
| HyperattentionDTI | 1.064± 0.080 | | 0.630± 0.013 | |  |
| PerceiverCPI | 0.658± 0.016 | | 0.675± 0.012 | |  |
| AiKPro | **0.399± 0.037** | | **0.810± 0.012** | |  |

**Table S6. Performance metrics comparison of AiKPro and AutoDock Vina at different cutoff values.**

| **Model** | **Cutoff** | **Precision** | **Recall** | **F1-score** | **AUC** | **Accuracy** |
| --- | --- | --- | --- | --- | --- | --- |
| AiKPro | 0.1 μM | 0.895 | **0.642** | **0.747** | **0.988** | **0.959** |
| AutoDock Vina |  | 0.328 | 0.415 | 0.367 | 0.764 | 0.865 |
| AiKPro | 1 μM | **1** | 0.205 | 0.341 | 0.9 | 0.739 |
| AutoDock Vina |  | 0.612 | 0.222 | 0.325 | 0.643 | 0.698 |
| AiKPro | 5 μM | **1** | 0.086 | 0.158 | 0.899 | 0.282 |
| AutoDock Vina |  | 0.970 | 0.147 | 0.256 | 0.674 | 0.327 |
